# Characterization of the Megavoltage Cone-Beam Computed Tomography (MV-CBCT) System on Halcyon^TM^ for IGRT: Image Quality Benchmark, Clinical Performance, and Organ Doses

**DOI:** 10.3389/fonc.2019.00496

**Published:** 2019-06-12

**Authors:** Irina Malajovich, Boon-Keng Kevin Teo, Heather Petroccia, James M. Metz, Lei Dong, Taoran Li

**Affiliations:** Department of Radiation Oncology, University of Pennsylvania, Philadelphia, PA, United States

**Keywords:** halcyon, MV-CBCT, IGRT, imaging dose, image quality

## Abstract

**Purpose:** The Varian Halcyon includes an ultrafast 6 MV flattening filter free (FFF) cone-beam computed tomography (MV-CBCT). Although a kV-CBCT add-on is available, in the basic configuration MV is used for image guided radiotherapy (IGRT). We characterized the MV-CBCT imager in terms of reproducibility, linearity, field of view (FOV) dependence, detectability of soft-tissue, and the effect of metal implants. The performance of the MV-CBCT in the clinic, including resulting dose to organs, is also discussed herein.

**Methods:** A Gammex phantom was scanned using a Halcyon MV-CBCT and a 120 kVp Siemens Definition Edge CT. Mean and standard deviation of Hounsfield Units (HUs) for different electron density relative to water ($\rho_{\text{e}}^{\text{W}}$) inserts were extracted. Doses to clinical patients due to MV-CBCT are calculated within Eclipse during treatment planning.

**Results:** A stable and near-linear HU-to-$\rho_{\text{e}}^{\text{W}}$ curve was obtained using the MV-CBCT. As the scan length increased from 10 to 28cm, the linearity of curve improved while the mean HUs decreased by 30%. All soft tissue inserts in the Gammex phantom were distinguishable. A crescent artifact affected HU measurements by up to 40 HUs. Soft-tissue contrast was sufficient for clinical online image-guidance in the low dose (5 MU) mode. Mean doses per fraction to organs-at-risk (OARs) were as high as 6 cGy for head and neck, 5 cGy for breast, and 4 cGy for pelvis patients. Metal rods did not affect HU values or introduce noticeable artifacts.

**Conclusions:** Halcyon's MV-CBCT has sufficient soft tissue contrast for IGRT and lacks metal-induced artifacts. Even though the absolute HU values vary with phantom size and scanning length, the HU-to-$\rho_{\text{e}}^{\text{W}}$ conversions are linear and stable day-to-day. In clinical cases, highest tissue doses from MV-CBCT ranged from 2-7cGy per fraction for various treatment sites, which could be significant for some organs at risk. Dose to out-of-treatment-field organs can be limited by reducing the scan length definition during planning and using the low dose mode. The high quality imaging mode did not provide material advantages over the low dose mode. Adequate IGRT was successfully delivered to multiple tumor sites using MV-CBCT.

## Introduction

The Halcyon system was designed as a vehicle for high-throughput image guided radiotherapy. This goal is achieved with a single energy linear accelerator (6 MV FFF) in an enclosed gantry, resulting in faster installation and commissioning, reduced cost of ownership, and streamlined workflows. For patient localization, the clinical beam is available for portal imaging and CBCT. The current Halcyon 2.0 version has an optional on-board kV-CBCT imager which has resulted in images comparable to diagnostic CTs (1). However, clinics looking to install a Halcyon may opt to forego the kV capabilities when the objective is to simplify and lower the cost of radiotherapy, or due to temporary unavailability to users in some regions. One of the main objectives of this study was to investigate the use of MV-CBCT in routine treatment using Halcyon's implementation.

MV imaging has been used for online IGRT applications (2, 3) and has advantages beyond financial considerations, such as having the same isocenter as the treatment beam and lacking metal artifacts. The latter advantage is well established (4), but had not been characterized in a Halcyon system yet. The main challenge for widespread use of MV imaging is the higher doses and lower image quality when compared to kV-based diagnostic equipment, and improvements in electronic portal imaging devices are a subject of current research (5, 6). Our institution was the first center to start treating patients using a Halcyon linear accelerator in September 2017. At that time, image guidance was achieved exclusively using the 6 MV FFF beam. In this report we characterize the quality and reproducibility of images obtained with the Halcyon on-board MV-CBCT, and review the clinical performance MV-CBCT for patient set-up, and organ dose distribution across various treatment sites.

## Materials and Methods

### Specifications of the Halcyon MV Imaging System

The Halcyon radiotherapy delivery system contains a single-energy X-band FFF linear accelerator, with 6 MV nominal X-ray energy. The linear accelerator is mounted on an enclosed gantry with a fixed opposing large format imaging panel at a source-to-imager distance of 154 cm, followed by a beam stopper. The imager dimensions are 43 ×43 cm with 0.336 mm pixel pitch in each dimension, and the maximum FOV at isocenter is 28 cm axial ×28 cm longitudinal with 0.22 mm pixel spacing. The panel is capable of operating at 25 frames/sec at full resolution to enable capturing at high dose rate without saturation. The field size is defined by a dual layer staggered multi-leaf collimator with leaves that project to a 1 cm width at isocenter. Although the FOV size in the axial plane is fixed at 28 cm, the longitudinal scan size can be adjusted during treatment planning. Selection of the longitudinal FOV is done by considering both the dose to the patient and the need to include required anatomical landmarks for alignment.

MV-CBCT imaging protocols on Halcyon take 15 s and are acquired with partial arc rotations from 260° to 100° in two dose settings: Low Dose (5 MU) and High Quality (10 MU). Daily MV imaging dose is not negligible and is always considered to contribute dose to targets and OARs. The imaging dose contribution is calculated within Eclipse with the same algorithm used for treatment dose calculations. At the time of this study, Eclipse (Varian Medical Systems, Palo Alto, CA) was the only treatment planning system available for Halcyon; care should be taken to incorporate MV-CBCT dose to the final total dose reviewed by the physician if a different treatment planning system is used. The Halcyon at our institution was calibrated to deliver 1cGy/MU with a 10 ×10 cm field, 100 SSD at dmax (1.3 cm depth).

### Phantom Measurements

A volumetric image of a Gammex 467 tissue characterization phantom (Sun Nuclear Corporation, Melbourne, FL) was used to characterize the MV-CBCT images. The phantom consists of a 33 cm diameter solid water disk with room for 16 1^′′^ rod inserts with different tissue equivalent electron densities. Additionally, a Catphan 504 (The Phantom Laboratory, Salem, NY) was used to further study the resulting image quality.

To provide reference CT (planning CT) images, a Siemens Somatom Definition Edge CT (Siemens Healthineers, Erlangen, Germany) was used to obtain helical images using abdomen settings (2.0 mm slices with B31s kernel, CTDIvol (32 cm) = 18.86 mGy, 120 kVp). For the studies with metal inserts, the extended HU setting was used and reconstruction was done with and without iterative metal artifact reduction (iMAR).

MV-CBCT of the phantoms were acquired using the two available protocols, High Quality (10 MU), and Low Dose (5 MU). To study dependence on FOV, acquisition was repeated for different longitudinal scanning length definitions. Acquisition was also repeated on different days to quantify HU stability as a function of time.

### Image Analysis

The phantom was scanned with both the diagnostic CT and the Halcyon MV-CBCT imagers. The mean Hounsfield Unit (HU) values and standard deviations were obtained by creating circular or cylindrical regions of interest in different phantom locations, and extracting their statistics using MIM software (MIM Software Inc., Beachwood, OH).

Tissues are evaluated in terms of their HU with respect to water (HU_tissue_−HU_water_), and images are characterized by the HU-to-$\rho_{\text{e}}^{\text{W}}$ response. The absolute HU of water in the Gammex phantom for MV-CBCT varies depending on the size of the phantom and scan length of the MV-CBCT. To decouple the global HU shift and relative HU differences across Gammex inserts, we analyzed data relative to water, i.e., we evaluated HU-to-$\rho_{\text{e}}^{\text{W}}$ with HU measurements relative to the true water insert inside the Gammex phantom.

With the exception of Contrast-to-Noise Metric (CN-Metric) studies, HU statistics are computed on cylindrical volumes consisting of 10 slices, 2 mm per slice, with a circular area of diameter 2.25 cm that contain approximately 3150 voxels. To evaluate the detectability of the Gammex soft tissue inserts using MV-CBCT images, we calculated CN-Metric from extracted average HUs and standard deviations using a single slice volume (circular area of diameter 2.25 cm containing ~315 voxels, 2 mm slice thickness).

### Organ Dose Analysis

Since imaging dose cannot be neglected, MV-imaging doses need to be calculated by the treatment planning system used. For Eclipse, Halcyon treatment plans incorporate MV-CBCT dose as part of the final dose distribution that physicians review and approve. The MV-CBCT dose is calculated with the same dose calculation algorithm as therapeutic beams on patient CTs, which enables direct visualization and analysis of organ specific dose distributions for various disease sites. The treatment planning algorithm was previously shown to accurately calculate doses delivered to a phantom (7). In this study we summarized key OAR doses for several disease sites most commonly treated using MV-CBCT for online IGRT in our clinic. Ten patients from each disease site were retrospectively analyzed and presented here.

## Results and Discussion

### MV-CBCT HU Response and Stability

**Figure 1:**
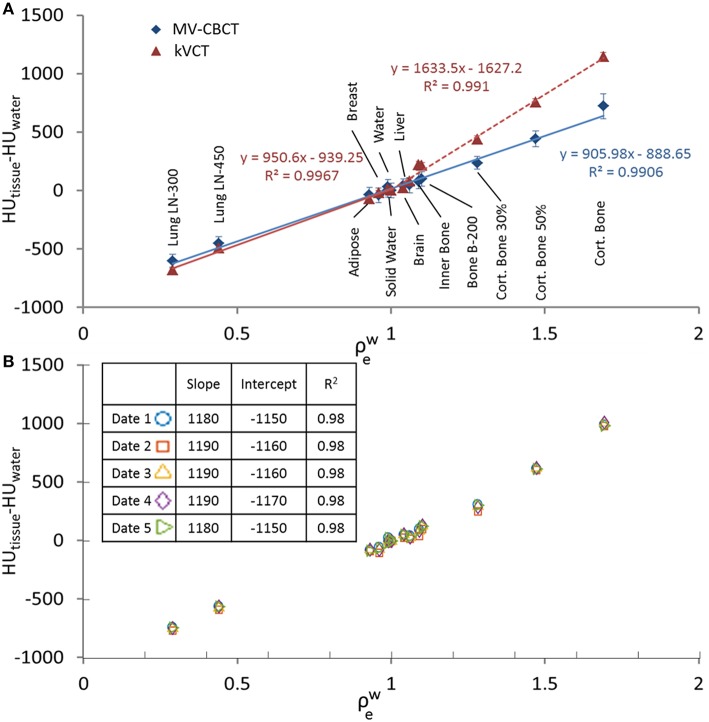
**(A)** HU_tissue_−HU_water_ vs. $\rho_{\text{e}}^{\text{w}}$ for MV-CBCT compared to kVCT with linear fittings, data taken with scan length = 24 cm. **(B)** HU_tissue_−HU_water_ vs. $\rho_{\text{e}}^{\text{w}}$ measured on different days. Data taken with scan length = 10 cm. The table insert in **(B)** shows the obtained slope and the coefficient of determination R^2^ when a linear regression is applied to the data.

A stable and near-linear relationship between HU and electron density relative to water (HU-to-$\rho_{\text{e}}^{\text{W}}$) was obtained using the Halcyon MV-CBCT. Whereas HU-to-$\rho_{\text{e}}^{\text{W}}$ is bi-linear for kVCT images (8), a single linear regression with R^2^ = 0.991 can be used for MV-CBCT images. This is shown in Figure 1A, where the difference between the average HU of a given tissue insert and the average HU of a water insert (HU_tissue_−HU_water_) in cylindrical volumes is plotted vs. the nominal $\rho_{\text{e}}^{\text{w}}$ for Gammex inserts. The MV-CBCT HU-to-$\rho_{\text{e}}^{\text{W}}$ response was reproducible, as shown in Figure 1B where data was collected on 5 different days in which the daily output varied as much as 1.3% as measured during the Machine Performance Check (9). The variation of the HU-to-$\rho_{\text{e}}^{\text{W}}$ response obtained from the measurements in Figure 1B is displayed as a table insert. Our results show that the HU response over time is stable within the noise of the images obtained.

### MV-CBCT HU Variability With Dose and Scan Length

**Figure 2:**
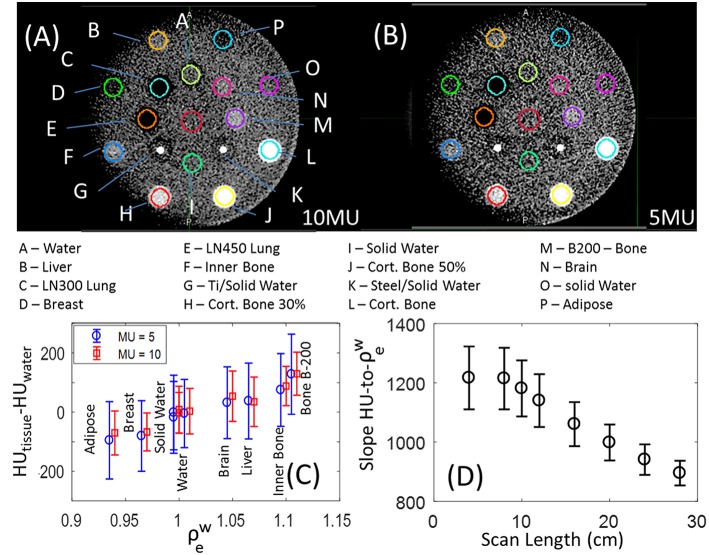
MV-CBCT of the Gammex phantom taken with the **(A)** high quality and **(B)** low dose setting, displayed with window width of 500 HU, and level of 50 HU. **(C)** HU values for the different soft tissue inserts for images acquired with the low (blue circles) and high (red squares); a small horizontal shift was added for clarity, and the error bars represent the statistical deviation of HUs in the volumes evaluated. **(D)** Slope to the linear fit of HU-to-$\rho_{\text{e}}^{\text{w}}$ as a function of longitudinal FOV, the error bars represent the 95% confidence interval.

Figures 2A,B show MV-CBCT images of the Gammex phantom in the high and low dose setting, respectively. Increasing the nominal dose from 5 to 10 MU reduced the noise by 30% without affecting the average HU values for different tissues, as shown in Figure 2C. Therefore, the HU-to-$\rho_{\text{e}}^{\text{W}}$ response is independent of the MV-CBCT dose. The low dose setting was found to be sufficient—and clinically preferred—for daily patient set-up.

Varying scan length (longitudinal FOV) has substantial impact on both the slope of the HU-to-$\rho_{\text{e}}^{\text{W}}$ as well as the linearity of the HU-to-$\rho_{\text{e}}^{\text{W}}$ fit. Figure 2D shows that as the scan length decreases from 28 to 8cm, the slope of HU-to-$\rho_{\text{e}}^{\text{W}}$ increases 36%, and the 95% confidence level error bars broaden by a factor of 2.5 indicating a linear fit is less accurate. The slope of HU-to-$\rho_{\text{e}}^{\text{W}}$ plateaus for scan lengths below 8 cm at 1200 HU/$\rho_{\text{e}}^{\text{W}}$, and decreases to 900 HU/$\rho_{\text{e}}^{\text{W}}$ for the maximum scan length size. The origin of the dependence of HU on scan length used is still under investigation. A calibration that mitigates the strong dependence of HU-to-$\rho_{\text{e}}^{\text{W}}$ on FOV is necessary for the implementation of Halcyon MV-CBCT-based dose calculations.

The data acquired for this study is limited to a single phantom with a fixed superior-inferior thickness and diameter. The dependence of results on the phantom size, which will likely impact both the HU and quality of MV-CBCT images, is left for future work.

### Image Artifacts

**Figure 3:**
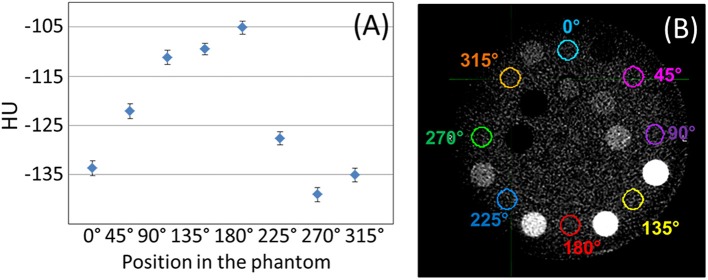
**(A)** Raw mean HU values measured on the solid water phantom, between inserts, for the positions shown in **(B)**, displayed with window width of 400 HU, and level at 40 HU.

A crescent artifact is visible in MV-CBCT images acquired by both of our two Halcyon units using the Gammex phantom. This artifact can be observed in Figures 2A,B, where the solid water frame of the Gammex looks darker on the left of the image than on the right. The low dose (5 MU) image B appears to be more impacted than the high dose (10 MU) image A. Previous literature suggested that this type of artifact may have originated from geometrical imperfections in a particular CBCT equipment of just 0.5 mm or 0.1° (10). The variation in HU with position caused by the crescent artifact can be quantified by measuring the HU statistics in the solid water frame of the Gammex, between the outer row of inserts (Figure 3). The solid water HU oscillates with angle about the Gammex in a sinusoidal-like fashion with a peak to valley variation of approximately 40 HUs around the edges of the phantom. The 40 HU variation due to the crescent artifact was also measured for the tissue equivalent inserts when the phantom was rotated. For online IGRT applications, this artifact does not generate significant impact, as users focuses more on local contrast for target/tissue alignment rather than global HU shifts. However, if MV-CBCT is to be used for treatment planning at a future time, the crescent artifact, if present, needs to be accounted for.

Another observation from Figure 3 is that the absolute HU values for solid water was not zero, but around −120 on average. In general, when evaluating the MV-CBCT images on Halcyon, we observed inconsistent HU shifts of water/solid water when the scatter conditions changed, e.g., different phantom sizes, different scanning length, and etc. For IGRT purpose this HU shift does not pose a significant challenge, however if the MV-CBCT image is used for dose calculations, careful calibration of the absolute HU for a particular phantom size and scanning length will be necessary.

### Soft Tissue Detectability and Spatial Resolution

**Figure 4:**
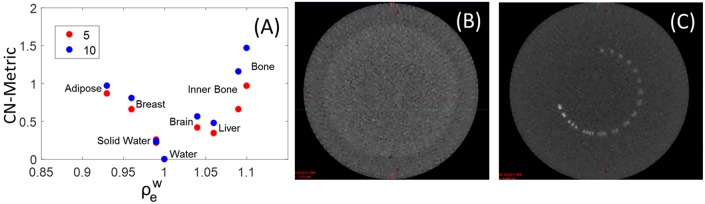
**(A)** Contrast to Noise Metric (CN-Metric) for soft tissue inserts in the Gammex phantom. Catphan high resolution **(C)** and low contrast sensitivity **(B)** images also shown with window width of 1,500 HU, and level at 600 HU.

The detectability of soft tissue inserts in a single MV-CBCT slice is shown in Figure 4A, where unlike previous figures statistics are not volumetric and are a representation of what a therapist would see at the console. The contrast-to-noise Metric (CN-Metric),

$$\begin{array}{l} \text{CN}-\text{Metric}=|{\text{HU}}_{\text{tissue}}-{\text{HU}}_{\text{sw}-\text{local}}|/\text{Std}.\text{Dev}._{\text{sw}-\text{local}} \end{array}$$

was calculated for each insert. A 40% on average increase in CN-Metric when dose increased from 5 to 10 MUs for soft tissue was obtained. To obtain a locally relevant measure of contrast in the presence of the crescent artifact, the mean HU of the water insert was scaled by the solid water surrounding each insert (HU_sw−local_). The denominator Std.Dev._water_ is still taken from actual water insert to be consistent. Although the Gammex soft tissue inserts were distinguishable from solid water, the low contrast region of a Catphan resulted in zero discernible circles (Figure 4B). The resolution pattern of the Catphan revealed that 4 line-pairs-per-cm can be visually resolved (Figure 4C). These findings are consistent with MV-CBCT studies in the literature (2, 11, 12).

### Clinical Examples of Halcyon MV-CBCT

**Figure 5:**
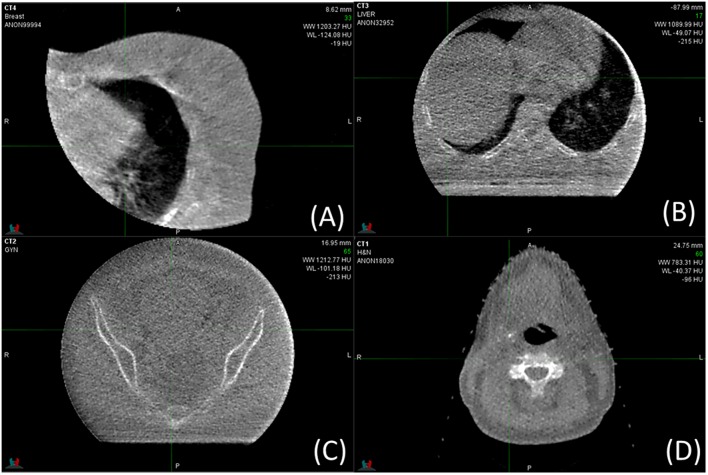
MV-CBCT images obtained for clinical treatments of different sites. Display parameters are: **(A)** WW: 1,203, WL: −124, **(B)** WW: 1,090, WL: −49, **(C)** WW: 1213, WL: −101, **(D)** WW: 783, WL: −40, (WW, window width; WL, window level).

Figure 5 shows clinical low dose (5 MU) MV-CBCT images for Breast (Figure 5A), Spine (Figure 5B), Pelvic (Figure 5C), and Head and Neck (Figure 5D) patients. Some of the tissues, such as muscle, adipose, bladder, rectum, bowel, heart, and liver, can be discerned in the clinical images. As a result, the on-board MV-CBCT in the Halcyon system was adequate for patient set-up. The crescent artifact is observed in Figures 5B,C but not in Figure 5D, showing that the size of the anatomy imaged has an impact on the severity of the artifact.

### OAR Dose From MV-CBCT

**Figure 6:**
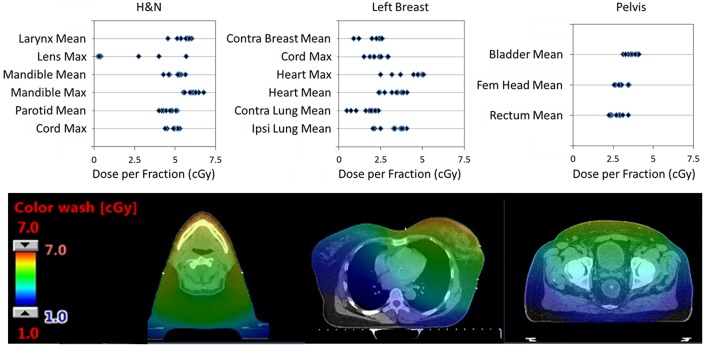
**(Top)** Single fraction dose to some OARs from a single MV-CBCT in the Low Dose setting for: head and neck (H&N), left breast, and pelvic patients. Results from 10 randomly selected patients is shown. **(Bottom)** Dose distributions from a typical patient used to extract OARs dose. All CT images are displayed with WW:350 and WL:50 (WW, window width; WL, window level).

In our Halcyon clinical implementation, we used MV-CBCT or MV-MV portals exclusively for patient set-up between September 2017 and July 2018. During this period, 190 patients were treated with Halcyon, 77 of them were set-up with daily MV-CBCT. Figure 6 shows the Eclipse-calculated dose contribution of MV-CBCT imaging to some of the critical organs for three different treatment sites: head and neck, left breast and pelvis. The corresponding dose distribution for representative cases for each site is also shown in Figure 6. The imaging isocenter was the same as the treatment isocenter, and images were acquired in the low dose (5 MU) setting with patients in the supine position. Doses resulting from imaging increased with decreasing patient separation, and were consistent with the values published by Li et al. for phantom measurements (7). For each site evaluated, dose distributions from 10 randomly selected patients were used to extract OAR doses.

Head and neck patients had masks for immobilization, and typically received treatment for 35 fractions. Resulting doses were highest on the mandibular bone and larynx due to their anterior location within the patient. Mean doses to the larynx, mandible, and parotids as well as maximum doses to the mandible, lenses and spinal cord are shown in Figure 6. The eye lenses were inside the imaging field for 3 patients, which resulted in doses as high as 2Gy (5.7cGy ×35 fractions) over the course of treatment. Since the MV-CBCT partial arc of the Halcyon is fixed and cannot be posterior to lower dose to the lenses (13), the superior edge of the imaging field of view should exclude the lenses when possible.

Breast patients had breast-boards for immobilization, and typically were treated with 16 fractions and additional 5 boost fractions. Due to large patient-to-patient anatomic variation, the distribution of doses to critical organs was broad for breast treatments. Doses to critical organs were higher for thin left breast patients with nodal involvement. Figure 6 shows results only for left laterality patients including chest wall, node involvement and breast reconstruction. Excluded from the figure are boost fraction MV-CBCTs which were typically smaller fields and resulted in lower doses to OARs. Figure 6 shows the mean doses to the contralateral breast, heart, contralateral lung and ipsilateral lung, and the maximum dose to the spinal cord and heart.

Patients in the pelvis site analysis of Figure 6 include: a prostate patient localized using fiducials and a rectal balloon, as well as gynecological and rectal/anal cancer patients localized with bony anatomy. Typical number of fractions was 28 for rectal/anal and gynecological patients, while the prostate patient received treatment in 32 fractions. Mean doses to the bladder, femoral head and rectum are shown in Figure 6.

It might be helpful to compare MV-CBCT doses relative to kV-CBCT, which are widely used for online IGRT purposes. Alaei et al. (14) summarized kV-CBCT doses in commercial linear accelerator systems available in 2015, and showed the per fraction dose measured ranged from less than 1 cGy for low dose head and neck imaging to 7 cGy and 2-3 cGy for pelvic protocols when measured on the skin and in the rectum, respectively. Deng et al. (15) reported for breast cancer patient undergoing kV-CBCT, average dose to heart, lungs, contralateral breast, and skin were 3.1, 3.2, 3.3, and 7-8 cGy per fraction, respectively. Based on these reported doses, the organ dose from MV-CBCT on Halcyon is comparable to dose from kV-CBCT for breast and pelvic imaging protocols, but substantially higher for head and neck IGRT applications.

One of the key limitation of the current implementation of MV-CBCT on Halcyon is the fixed arc geometry from 100° to 260° (or reversed). The length of the arc, as well as the start and stop angles, are not customizable by the user. This increases dose to the anteriorly located OARs, such as the lenses and the heart. A posterior arc could help reduce OAR dose in these cases (13). We have made the suggestion to the vendor to allow users define start and stop angles of the MV-CBCT acquisition, and hope to see this limitation addressed in future releases of the product.

### Impact From Metal Hardware on Image Quality

**Figure 7:**
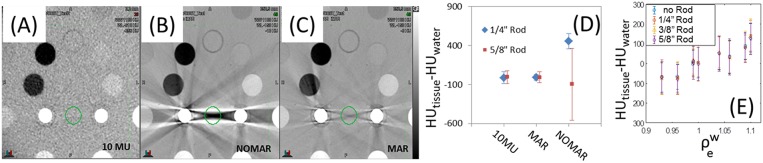
Gammex phantom images from **(A)** MV-CBCT, **(B)** CT without metal artifact reduction (MAR) and **(C)** CT with MAR. The resulting average HU and standard deviation between two rods is shown in **(D)**, and the invariance of HU with metal rod size in **(E)**. Display parameters are: **(A)** WW: 1,100, WL−310, **(B)** WW: 1,100, WL−150, **(C)** WW: 1,100, WL−150. (WW, window width; WL, window level).

The effect metal rod inserts have on image quality can be seen in Figure 7, where the metal-induced artifacts are: not observed in the MV-CBCT (Figure 7A), and severe for kVCT images (Figure 7B). The Siemens CT scanner allows for metal artifact reduction (MAR), shown in Figure 7C where artifacts are diminished but still present. A quantification of the artifacts is obtained by analyzing the HU variation within a cylindrical volume between two rods shown in green in Figure 7D. As the volume of the implant increases, MAR fails to fully correct artifacts. Though it would appear from Figure 7D that the MAR and 10 MU images are similar for larger rods, the variation in HU for the 10 MU data is random whereas the variation in HU for the MAR corrected data is systematic (and is dependent on location of measurement). The MV-CBCT images approach HU saturation for steel as the FOV is decreased from 20 cm, allowing for differentiation between medically relevant metal inserts. For example, the HU values obtained for steel and titanium were 7000 and 3000, respectively, for the 28 cm FOV setting. HU values increase with decreasing FOV and start to saturate when the longitudinal size is 10 cm or less. The average-HUs for soft tissue equivalent inserts obtained with MV-CBCT did not depend on the presence or size of the metal implant (Figure 7E). Given the lack of metal artifacts and the ability to distinguish metal implants, MV-CBCT could be a convenient imaging method for planning quick turn-around cases in an emergency setting (16), or could be used to supplement a kVCT used for planning for patients with significant metal implants (17–20).

### Study Limitations

There are several limitation of the current study that should be noted. The study was conducted on a single Halcyon unit over a short period of times. The phantom used in this study was a fixed size phantom, therefore we were not able to test image quality variations as a function of phantom size. We are currently performing data collection and evaluation of MV-CBCT quality across multiple Halcyon units over an extended period of time. Additional studies to assess HU variability with phantom size are also underway. We hope to report these results soon.

## Conclusions

Halcyon MV-CBCT images are capable of discerning different soft tissues and lack metal-induced artifacts. Although the absolute HU values varies as scatter condition changes, e.g., phantom size and/or scanning length, HU-to-$\rho_{\text{e}}^{\text{W}}$ conversions were linear and stable day-to-day, but still showed scan length dependence and presence of a crescent artifact which should be considered if CBCT images are used for dose calculation. In clinical cases, highest tissue doses from MV-CBCT ranged from 2-7 cGy per fraction for various treatment sites, which is comparable to per fraction dose from kV CBCTs during breast and pelvic IGRT applications, but substantially higher than low dose head and neck protocol. Dose to out-of-treatment-field organs can be limited by reducing the scan length definition during planning and using the low dose mode. We found the high quality 10 MU imaging mode did not provide material advantages over the low dose 5 MU mode for IGRT in the clinic.

Although Varian now offers kV-CBCT as an add-on for Halcyon systems, MV-CBCT may still be the preferred imaging technique when patients have significant implanted metal and when institutions cannot afford the kV upgrade, such as in the developing world. The dose from MV-CBCT imaging is calculated by the treatment planning system and incorporated into plans for patients. Physicians visualize, review, and approve plans that account for MV-CBCT imaging dose, which is tracked throughout the treatment course together with the therapeutic dose. Despite the described MV-CBCT limitations and additional dose to OARs, high quality IGRT treatment plans were successfully delivered for multiple tumor sites.

## Ethics Statement

This retrospective study was carried out in accordance with the recommendations of University of Pennsylvania, Internal Review Board. The protocol and waiver of consent was approved by the University of Pennsylvania IRB (#829182).

## Author Contributions

IM helped design the experiments, acquired most of and analyzed all the data, and drafted a first manuscript version. HP helped perform some of the experiments. TL conceived of the study, helped design experiments, and helped to finalize the manuscript. BT helped design experiments. BT, JM, LD, and TL contributed with regard to scientific context and financial or technical support. All authors helped with the interpretation of data, revised the manuscript critically, and approved the final manuscript.

### Conflict of Interest Statement

TL reports personal fees from Varian Medical Systems unrelated to this project. LD reports personal fees and grants from Varian Medical Systems that contributed to the project. JM is on an advisory board of Varian Medical Systems, and received a grant that contributed to this project. The remaining authors declare that the research was conducted in the absence of any commercial or financial relationships that could be construed as a potential conflict of interest.

## References

[1] Cai B Mazur T Laugeman E Park J Hugo G Mutic S Characterization of a prototype rapid on-board KV imaging system designed for a ring shape radiation therapy unit. Med Phys. (2018) 45:E525 10.1002/mp.13396 PMC8188470 30675902

[2] Meeks SL Harmon JF Jr Langen KM Willoughby TR Wagner TH Kupelian PA. Performance characterization of megavoltage computed tomography imaging on a helical tomotherapy unit. Med Phys. (2005) 32:2673–81. 10.1118/1.1990289 16193798

[3] Held M Cremers F Sneed PK Braunstein S Fogh SE Nakamura J. Assessment of image quality and dose calculation accuracy on kV CBCT, MV CBCT, and MV CT images for urgent palliative radiotherapy treatments. J Appl Clin Med Phys. (2016) 17:279–90. 10.1120/jacmp.v17i2.6040 27074487 PMC5874969

[4] Ruchala KJ Olivera GH Schloesser EA Mackie TR. Megavoltage CT on a tomotherapy system. Phys Med Biol. (1999) 44:2597–621. 10.1088/0031-9155/44/10/316 10533931

[5] Hu YH Rottmann J Fueglistaller R Myronakis M Wang A Huber P. Leveraging multi-layer imager detector design to improve low-dose performance for megavoltage cone-beam computed tomography. Phys Med Biol. (2018) 63:035022. 10.1088/1361-6560/aaa160 29235440 PMC5824638

[6] Myronakis M Hu YH Fueglistaller R Wang A Baturin P Huber P. Multi-layer imager design for mega-voltage spectral imaging. Phys Med Biol. (2018) 63:105002. 10.1088/1361-6560/aabe21 29652670 PMC5991631

[7] Li Y Netherton T Nitsch PL Balter PA Gao S Klopp AH. Normal tissue doses from MV image-guided radiation therapy (IGRT) using orthogonal MV and MV-CBCT. J Appl Clin Med Phys. (2018) 19:52–7. 10.1002/acm2.12276 29500856 PMC5978715

[8] Constantinou C Harrington JC DeWerd LA. An electron density calibration phantom for CT-based treatment planning computers. Med Phys. (1992) 19:325–7. 10.1118/1.596862 1584125

[9] Li Y Netherton T Nitsch PL Gao S Klopp AH Balter PA. Independent validation of machine performance check for the Halcyon and TrueBeam linacs for daily quality assurance. J Appl Clin Med Phys. (2018) 19:375–82. 10.1002/acm2.12391 30016578 PMC6123154

[10] Giles W Bowsher J Li H Yin FF. Crescent artifacts in cone-beam CT. Med Phys. (2011) 38:2116–21. 10.1118/1.3567508 21626944

[11] Chan M Yang J Song Y Burman C Chan P Li S. Evaluation of imaging performance of major image guidance systems. Biomed Imaging Interv J. (2011) 7:e11. 10.2349/biij.7.2.e11 22287985 PMC3265149

[12] Gayou O Miften M. Commissioning and clinical implementation of a mega-voltage cone beam CT system for treatment localization. Med Phys. (2007) 34:3183–92. 10.1118/1.2752374 17879781

[13] Ding GX Munro P. Radiation exposure to patients from image guidance procedures and techniques to reduce the imaging dose. Radiother Oncol. (2013) 108:91–8. 10.1016/j.radonc.2013.05.034 23830468

[14] Alaei P Spezi E. Imaging dose from cone beam computed tomography in radiation therapy. Phys Med. (2015) 31:647–58. 10.1016/j.ejmp.2015.06.003 26148865

[15] Deng J Chen Z Knisely J Nath R Kilo-voltage imaging doses to organs-at-risk in cbct-guided breast radiation therapy. Int J Radiat Oncol Biol Phys. (2012) 84:S233–4. 10.1016/j.ijrobp.2012.07.606

[16] Held M Sneed PK Fogh SE Pouliot J Morin O. Feasibility of MV CBCT-based treatment planning for urgent radiation therapy: dosimetric accuracy of MV CBCT-based dose calculations. J Appl Clin Med Phys. (2015) 16:458–71. 10.1120/jacmp.v16i6.5625 26699575 PMC5690985

[17] Veiga C Janssens G Teng CL Baudier T Hotoiu L McClelland JR. First Clinical investigation of cone beam computed tomography and deformable registration for adaptive proton therapy for lung cancer. Int J Radiat Oncol Biol Phys. (2016) 95:549–59. 10.1016/j.ijrobp.2016.01.055 27084664

[18] Aubin M Morin O Chen J Gillis A Pickett B Aubry JF. The use of megavoltage cone-beam CT to complement CT for target definition in pelvic radiotherapy in the presence of hip replacement. Br J Radiol. (2006) 79:918–21. 10.1259/bjr/19559792 16916807

[19] Onozato Y Kadoya N Fujita Y Arai K Dobashi S Takeda K. Evaluation of on-board kV cone beam computed tomography-based dose calculation with deformable image registration using Hounsfield unit modifications. Int J Radiat Oncol Biol Phys. (2014) 89:416–23. 10.1016/j.ijrobp.2014.02.007 24685445

[20] Gao LG Sun HF Ni XY Fang MM Cao Z Lin T Metal artifact reduction through MVCBCT and kVCT in radiotherapy. Sci Rep. (2016) 6:37608 10.1038/srep37608 27869185 PMC5116646

